# Supplementary material for: Iterative improvement in the automatic modular design of robot swarms
Source: PeerJ Comput Sci. 2020 Dec 7;6:e322. doi: 10.7717/peerj-cs.322 (PMC7924708; doi:10.7717/peerj-cs.322)
Supplement: Supplemental Information 3 [file peerj-cs-06-322-s003.zip › argos3/doc/api/standalone/a00545_source.html]

ARGoS: plugins/robots/generic/simulator/range\_and\_bearing\_medium\_sensor.h Source File


- Main Page
- Related Pages
- Namespaces
- Classes
- Files

- File List
- File Members

# plugins/robots/generic/simulator/range\_and\_bearing\_medium\_sensor.h

Go to the documentation of this file.

```
00001 
00007 #ifndef RANGE_AND_BEARING_MEDIUM_SENSOR_H
00008 #define RANGE_AND_BEARING_MEDIUM_SENSOR_H
00009 
00010 #include <string>
00011 #include <map>
00012 
00013 namespace argos {
00014    class CRangeAndBearingMediumSensor;
00015    class CRABEquippedEntity;
00016    class CControllableEntity;
00017    class CRABMedium;
00018 }
00019 
00020 #include <argos3/core/simulator/sensor.h>
00021 #include <argos3/core/utility/math/rng.h>
00022 #include <argos3/core/simulator/space/space.h>
00023 #include <argos3/plugins/robots/generic/control_interface/ci_range_and_bearing_sensor.h>
00024 
00025 namespace argos {
00026 
00027    class CRangeAndBearingMediumSensor : public CSimulatedSensor,
00028                                         public CCI_RangeAndBearingSensor {
00029 
00030    public:
00031 
00032       CRangeAndBearingMediumSensor();
00033       virtual ~CRangeAndBearingMediumSensor() {}
00034       virtual void SetRobot(CComposableEntity& c_entity);
00035       virtual void Init(TConfigurationNode& t_tree);
00036       virtual void Update();
00037       virtual void Reset();
00038       virtual void Destroy();
00039 
00040    private:
00041 
00042       CRABEquippedEntity*  m_pcRangeAndBearingEquippedEntity;
00043       CControllableEntity* m_pcControllableEntity;
00044       CRABMedium*          m_pcRangeAndBearingMedium;
00045       Real                 m_fDistanceNoiseStdDev;
00046       Real                 m_fPacketDropProb;
00047       CRandom::CRNG*       m_pcRNG;
00048       CSpace&              m_cSpace;
00049       bool                 m_bShowRays;
00050    };
00051 }
00052 
00053 #endif
```

---

Generated on 10 Jul 2018 for ARGoS by 
 1.6.1
